# Supplementary material for: Cross-expression meta-analysis of mouse brain slices reveals coordinated gene expression across spatially adjacent cells
Source: Genome Biol. 2025 Oct 29;26:373. doi: 10.1186/s13059-025-03747-8 (PMC12570847; doi:10.1186/s13059-025-03747-8)
Supplement: Supplementary file 1 — Additional file 1. Supplementary Fig. S1-S10 contains the supplementary figures. [file 13059_2025_3747_MOESM1_ESM.docx]

Supplementary Figures S1-S10

Cross-expression meta-analysis of mouse brain slices reveals coordinated gene expression across spatially adjacent cells

Ameer Sarwar^1^ ([ameer.sarwar@mail.utoronto.ca](mailto:ameer.sarwar@mail.utoronto.ca)), Mara Rue^2^ ([mara.rue@alleninstitute.org](mailto:mara.rue@alleninstitute.org)) Leon French^3^ ([leon.french@utoronto.ca](mailto:leon.french@utoronto.ca)), Helen Cross^2^ ([crossh@carleton.edu](mailto:crossh@carleton.edu)), Sarah Choi^3^ ([sr.choi@mail.utoronto.ca](mailto:sr.choi@mail.utoronto.ca)), Xiaoyin Chen^2^ ([xiaoyin.chen@alleninstitute.org](mailto:xiaoyin.chen@alleninstitute.org)) and Jesse Gillis^3^* ([jesse.gillis@utoronto.ca](mailto:jesse.gillis@utoronto.ca))

^1^Department of Cell and Systems Biology and Donnelly Centre for Cellular and Biomolecular Research, University of Toronto, Toronto, ON, Canada

^2^Allen Institute for Brain Science, Seattle, WA, USA

^3^Department of Physiology and Donnelly Centre for Cellular and Biomolecular Research, University of Toronto, Toronto, ON, Canada

*Corresponding Author

**Fig. S1**


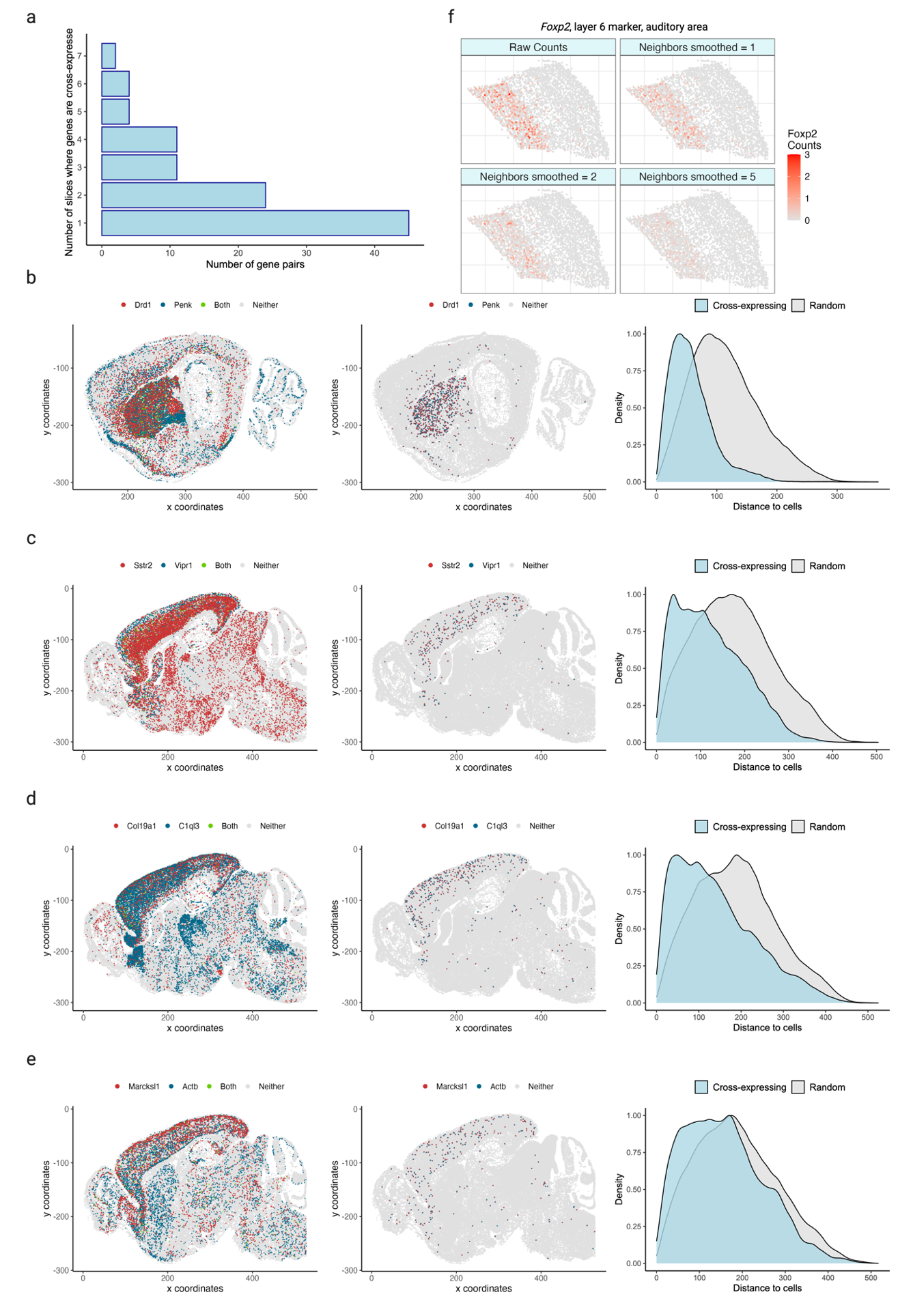


**Fig. S1. Cross-expression across tissue slices and regions for ligand-receptor and non-signaling genes. a**, Distribution of the number of gene pairs cross-expressed in different slices. Dataset has 16 slices sampled sagittally from the left hemisphere of a mouse brain. **b-e**, Cells are colored by gene expression (left) and cross-expressing cells are highlighted (center). Right, distances between cross-expressing cells are compared with those between cross-expressing and randomly selected cells. Smaller distances mean that cross-expressing cells are nearer each other (spatial enrichment) than expected by chance (p-values ≤ 0.01, left-tailed Mann-Whitney U test). Genes include ligands and receptors (b, c) and non-ligands and non-receptors (d, e). **f**, Smoothed gene expression for different numbers of neighbors for the auditory cortical layer 6 marker gene *Foxp2*. Created with BioRender.com.

**Fig. S2**


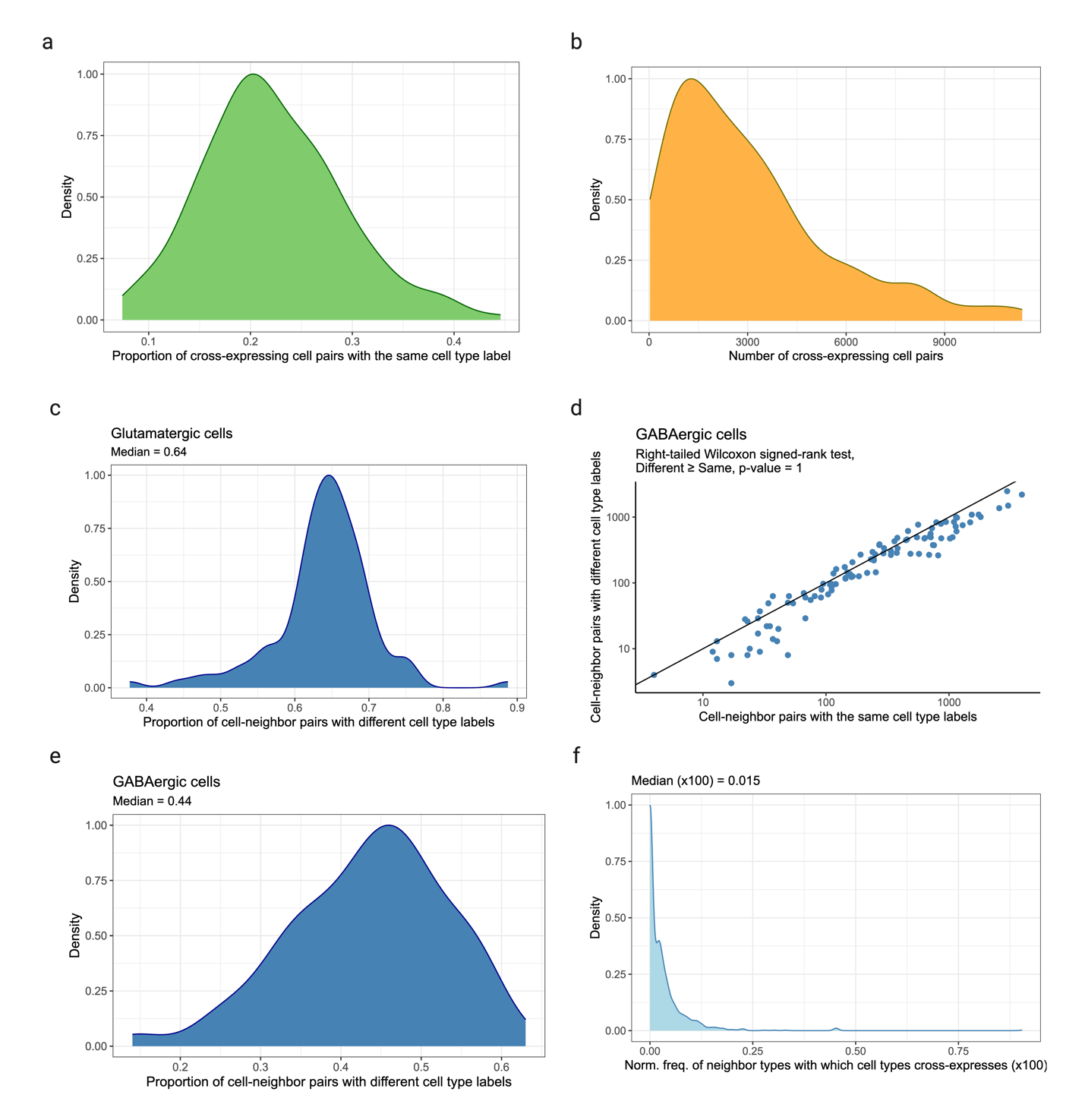


**Fig. S2. Relationship between cross-expression and cell type heterogeneity. a**, Proportion of cross-expressing cell pairs belonging to the same cell type label. **b**, Number of cell-neighbor pairs involved in cross-expression. **c**, Proportion of cell-neighbor pairs with different cell subtype labels given that both were labeled ‘glutamatergic’ at the higher level in the cell type hierarchy. **d**, Number of cell-neighbor pairs with the same or different cell subtype label given that both were labeled ‘GABAergic’ at the higher level in the cell type hierarchy. Each point is a cross-expressing gene pair. **e**, Same as in (c) but for ‘GABAergic’ cells. **f**, Proportion of neighbor cell types against which cell types cross-express. Created with BioRender.com.

**Fig. S3**


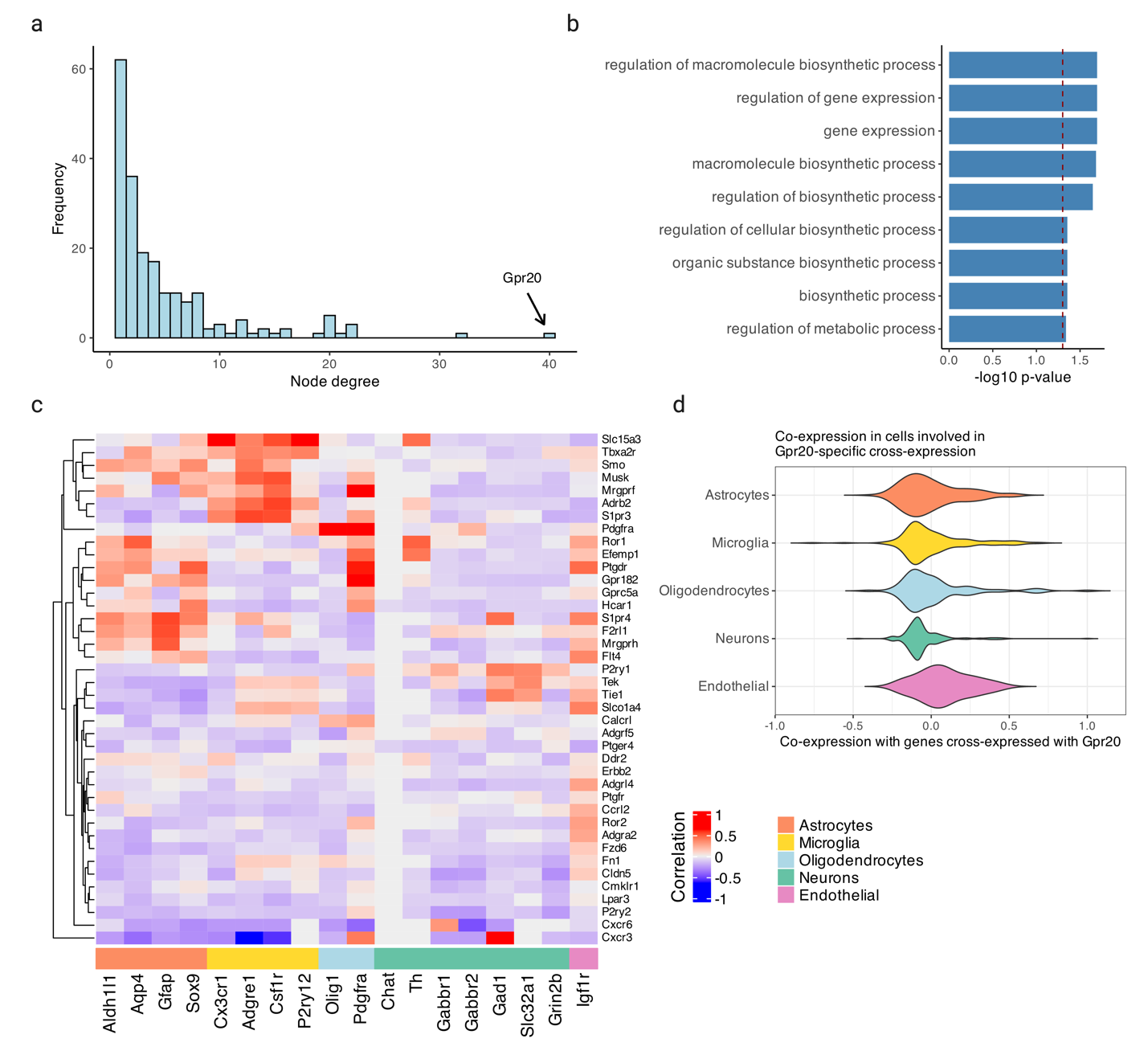


**Fig. S3. Exploration of *Gpr20* and its cross-expressing genes. a**, Distribution of node degree, with *Gpr20* highlighted. **b**, Gene ontology (GO) functional groups for genes cross-expressed with *Gpr20*. **c**, Co-expression of genes cross-expressed with *Gpr20* (right) against cell type marker genes (bottom). For each gene, co-expression was computed using cells involved in cross-expression and not the entire dataset. **d**, Distribution of cell type marker genes’ co-expression across the genes in (c). Created with BioRender.com.

**Fig. S4**


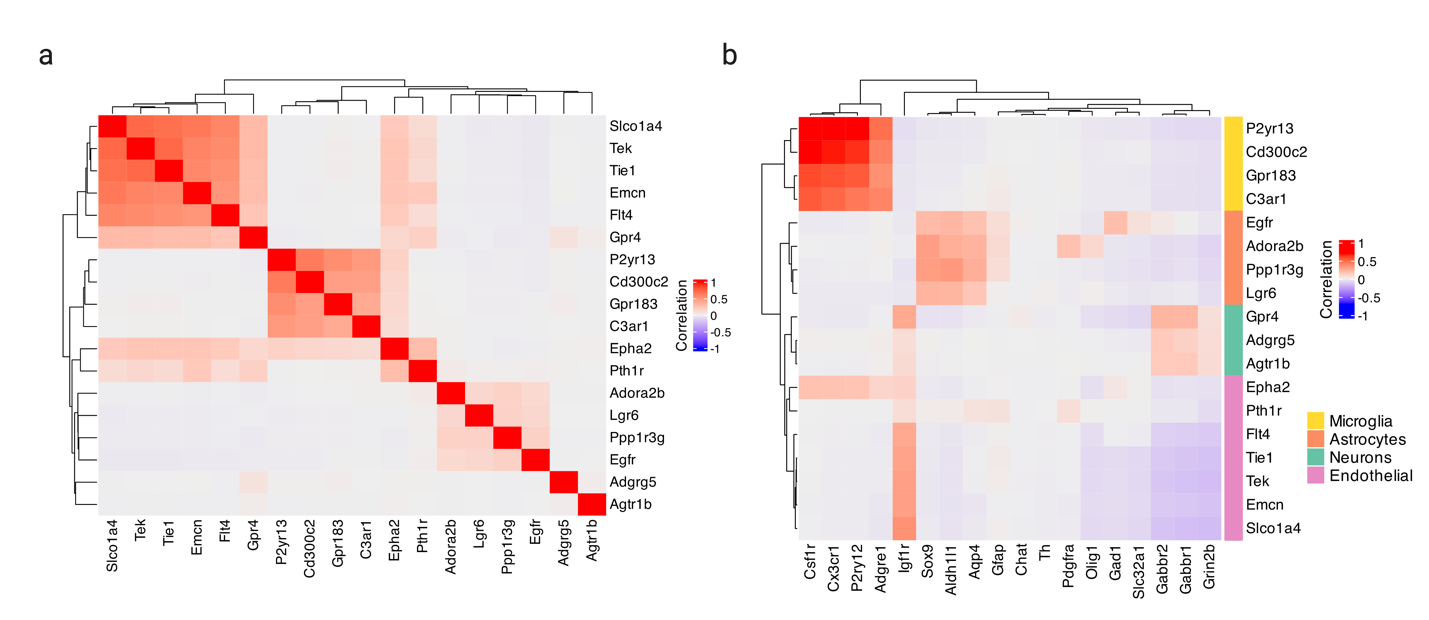


**Fig. S4. Exploration of the MERFISH cross-expression (sub)network. a**, Co-expression of genes in the subnetwork. **b**, Co-expression between genes in the subnetwork (right) and cell type marker genes (bottom). Created with BioRender.com.

**Fig. S5**


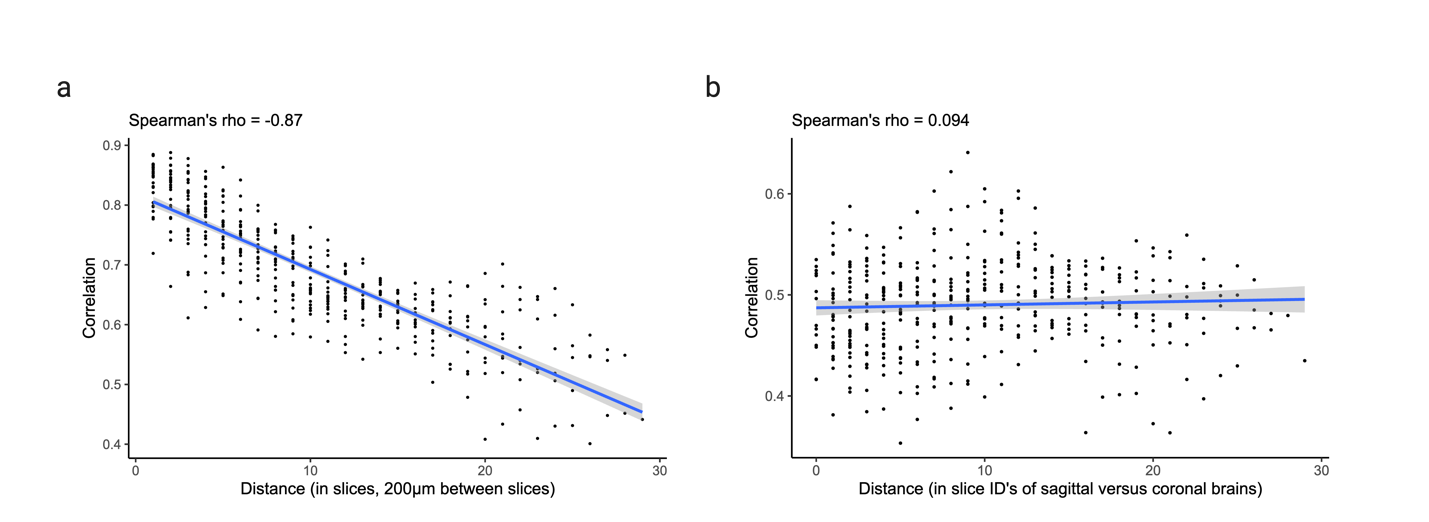


**Fig. S5. Cross-expression network similarity between slices. a**, Slice-specific cross-expression networks compared and shown as a function of distance between slices. **b**, Same as in (a) but slice-specific networks compared between sagittal and coronal datasets, where the “distance” is the difference in slice ID's. Shaded areas are 95% confidence intervals. Created with BioRender.com.

**Fig. S6**


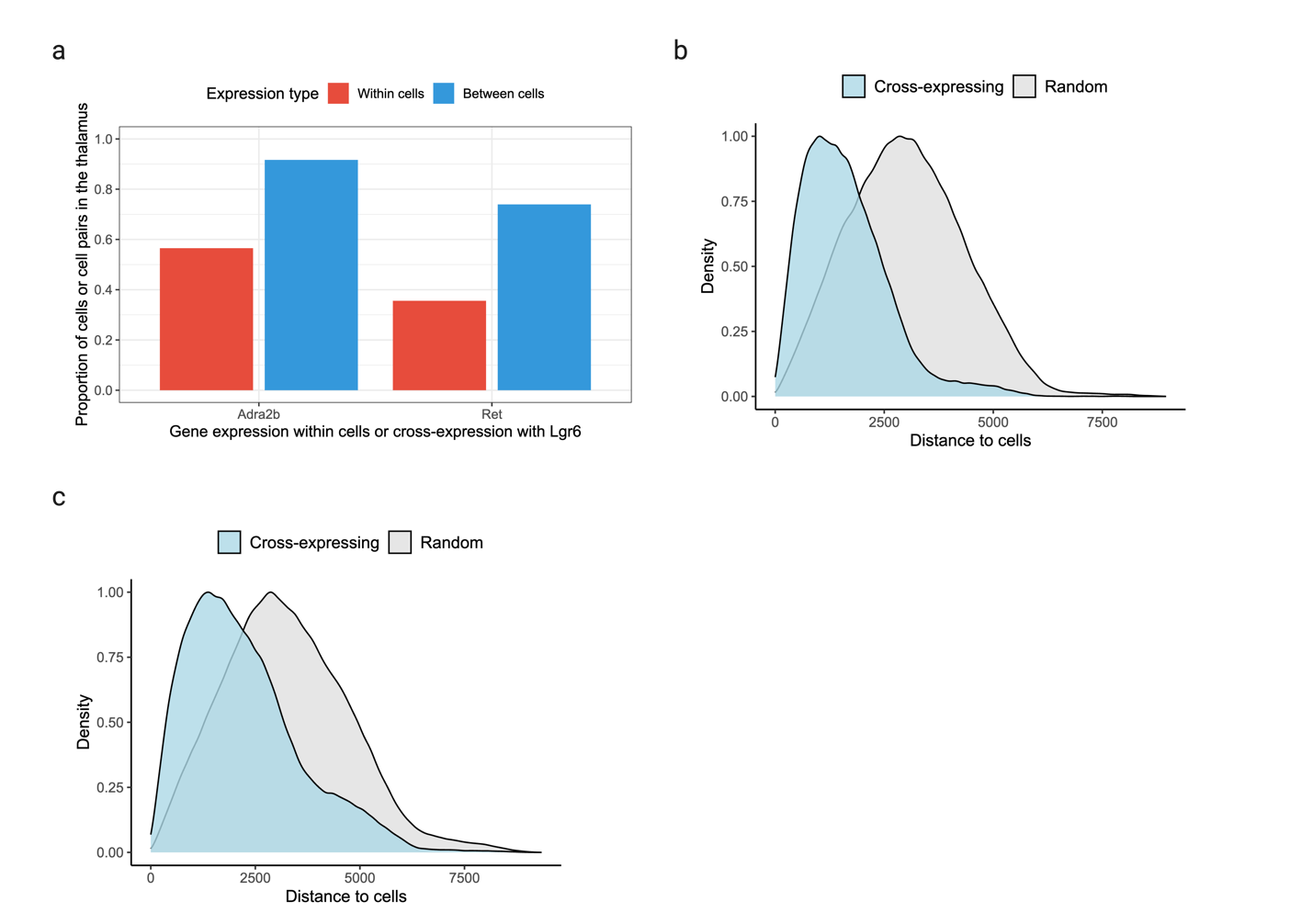


**Fig. S6. Combinatorial anatomical markers discovered using spatially enriched cross-expression. a**, Proportion of *Adra2b*- and *Ret*-expressing cells in the thalamus (red) and the proportion of cell pairs in the thalamus (blue) when cross-expressing with *Lgr6*. **b-c**, Distances between cross-expressing cells versus those between cross-expressing and randomly chosen cells for genes *Lgr6* and *Adra2b* (c) and for *Lgr6* and *Ret* (d). Smaller distances mean that cross-expressing cells are nearer each other (spatial enrichment) than expected by chance (p-values ≤ 0.01, left-tailed Mann-Whitney U test). Created with BioRender.com.

**Fig. S7**


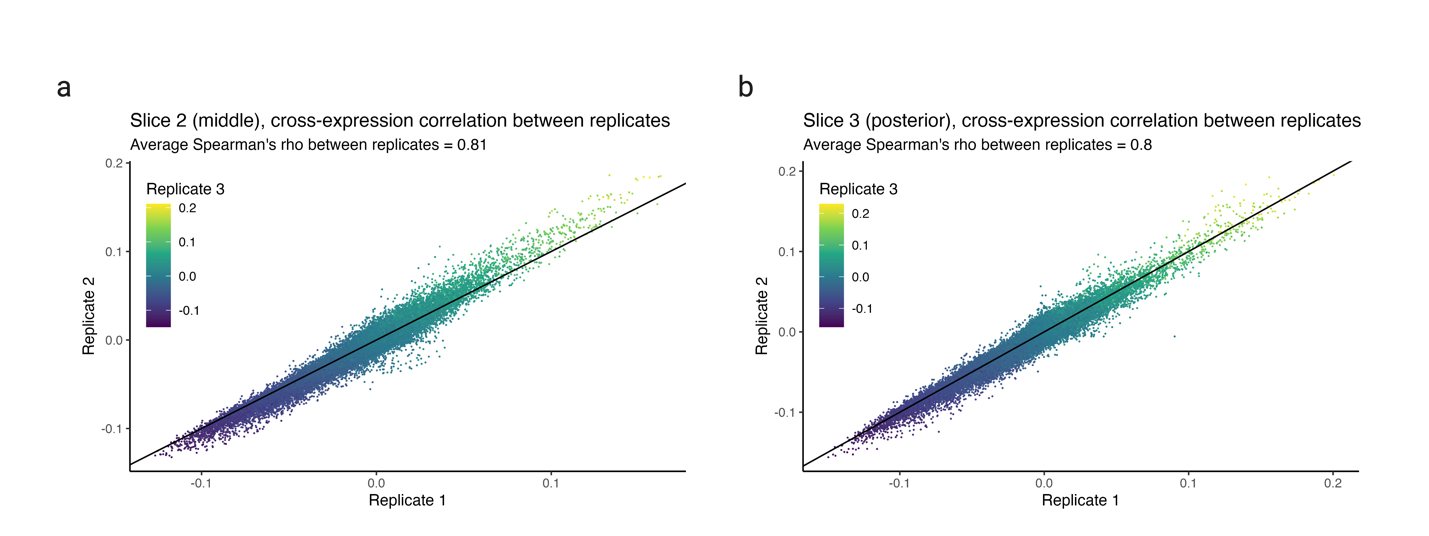


**Fig. S7. Cross-expression network similarity between replicates. a-b**, Cross-expression networks compared between three replicates for the middle (a) and posterior (b) slices. Created with BioRender.com.

**Fig. S8**


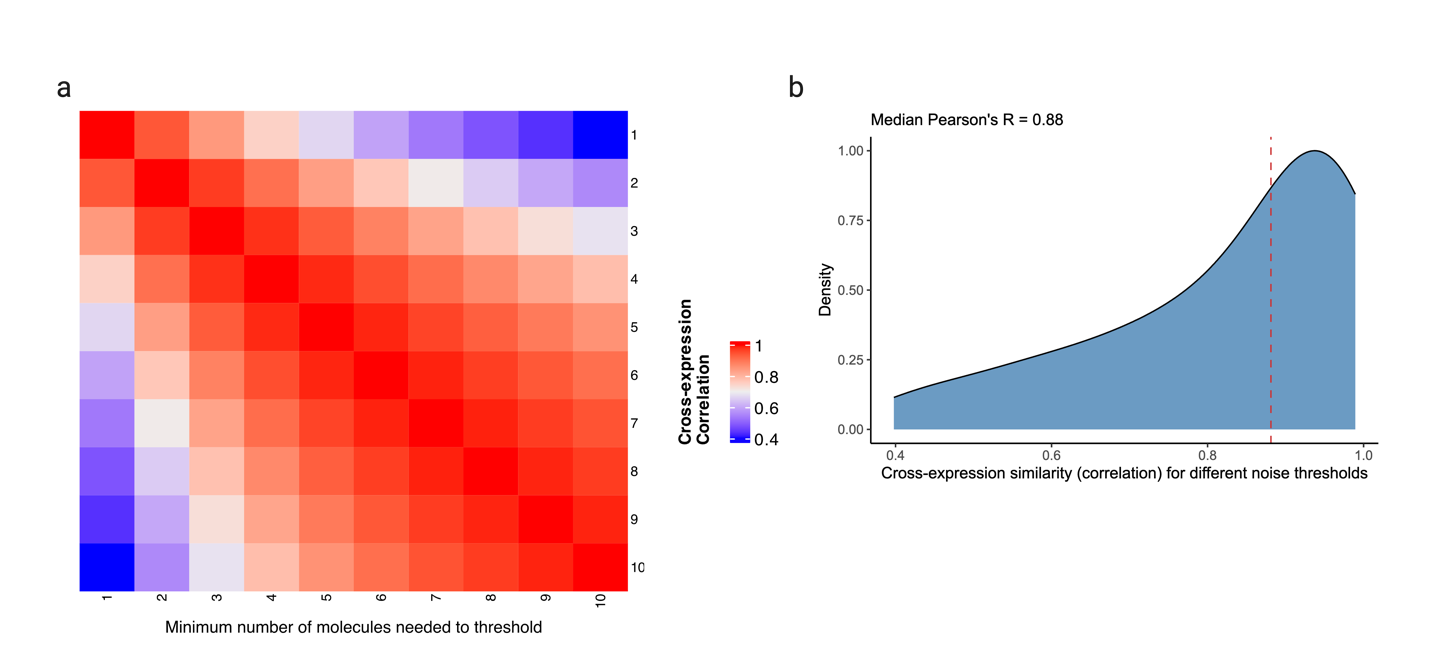


**Fig. S8. Cross-expression network similarity at different levels of gene expression noise thresholds.** **a**, Cross-expression networks compared after applying different noise thresholds, which are the minimum number of molecules a gene must express within a cell to be considered as detected. **b**, Distribution of the network similarities across noise levels, with the median indicated using the dotted line. Created with BioRender.com.

**Fig. S9**


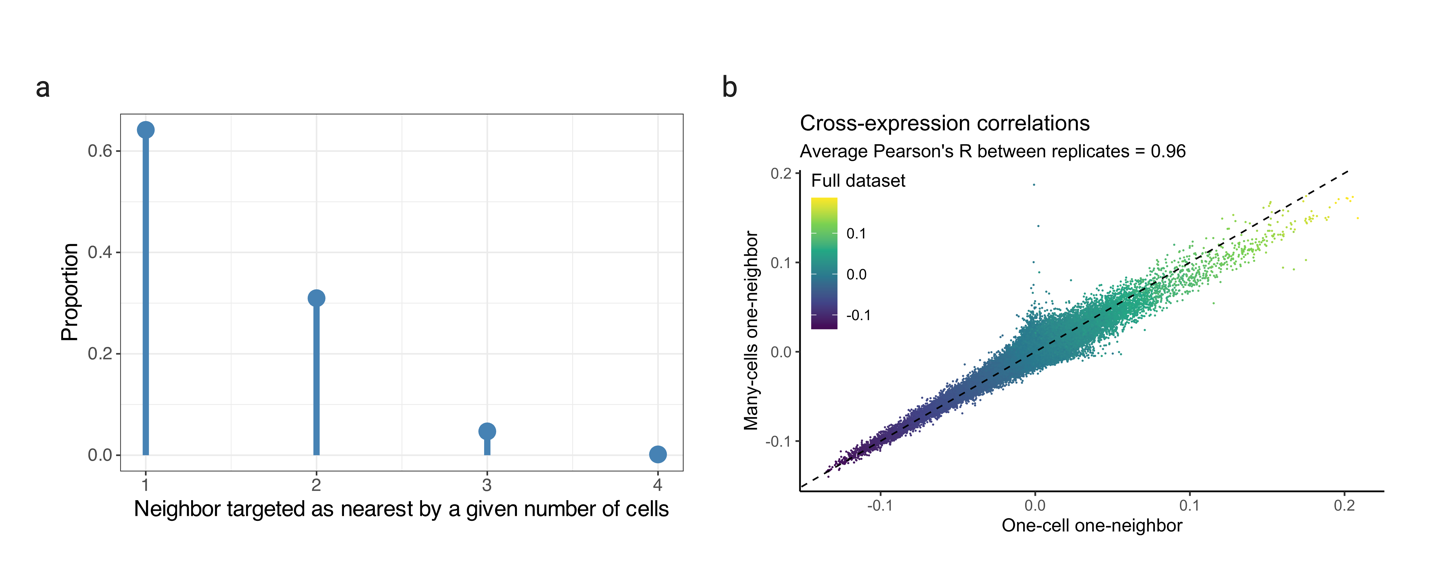


**Fig. S9. Patterns of cell-neighbor mappings and their relationship with cross-expression.** **a**, Cells considered as ‘nearest neighbor’ by other cells reported as a proportion of total cell-neighbor relations. ‘1’ is one-to-one mapping and ‘2-4’ is many-to-one mapping. **b**, Cross-expression networks computed using one-to-one mappings, many-to-one mappings, and the full dataset (both mappings). Created with BioRender.com.

**Fig. S10**

**
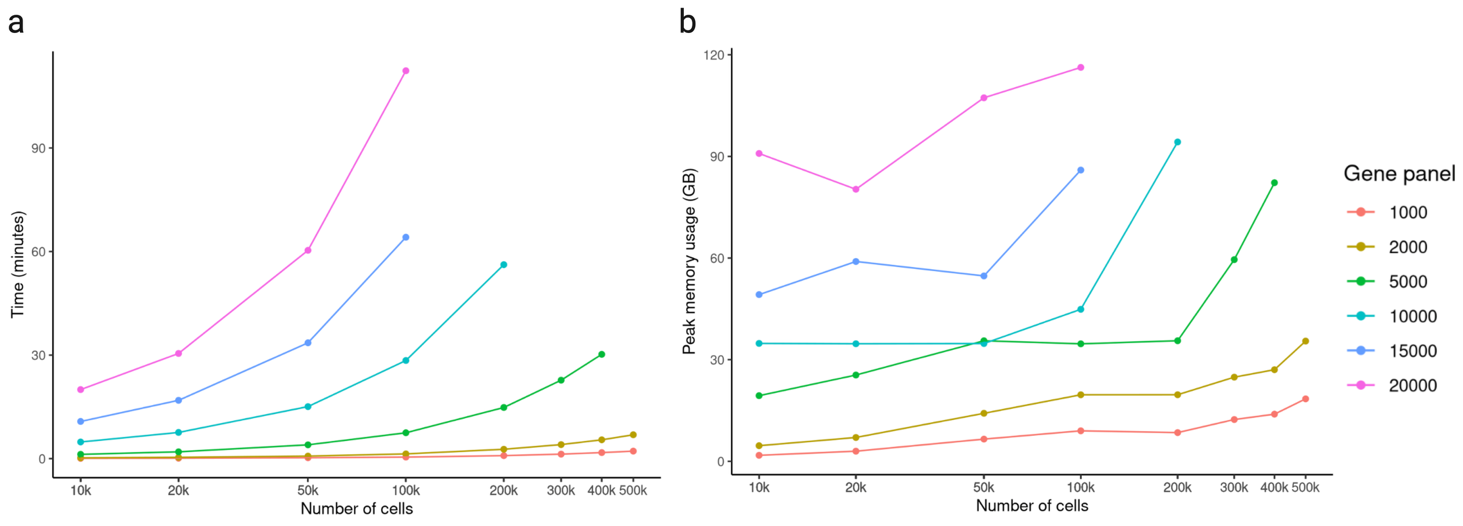
**

**Fig. S10. Computational efficiency of the R software.** Time (a) and memory (b) requirements of computing cross-expression for all gene pairs across different number of cells and gene panel sizes. Created with BioRender.com.
